# The impact of diabetes mellitus on the emergence of multi-drug resistant tuberculosis and treatment failure in TB-diabetes comorbid patients: a systematic review and meta-analysis

**DOI:** 10.3389/fpubh.2023.1244450

**Published:** 2023-11-16

**Authors:** Anees ur Rehman, Mahnoor Khattak, Usman Mushtaq, Muhammad Latif, Imran Ahmad, Muhammad Fawad Rasool, Sadia Shakeel, Khezar Hayat, Rabia Hussain, Ghaidaa Ali Alhazmi, Afnan Owedah Alshomrani, Mohammed Ibrahim Alalawi, Saleh Alghamdi, Mohammad Tarique Imam, Safa S. Almarzoky Abuhussain, Sarah M. Khayyat, Abdul Haseeb

**Affiliations:** ^1^Department of Pharmacy Practice, Faculty of Pharmacy, Bahauddin Zakariya University, Multan, Pakistan; ^2^Nishter Medical University and Hospital, Multan, Pakistan; ^3^Department of Zoology, Division of Science and Technology, University of Education Lahore, Lahore, Pakistan; ^4^Department of Pharmaceutical Chemistry, Faculty of Pharmacy, Bahauddin Zakariya University, Multan, Pakistan; ^5^Department of Pharmacy Practice, Faculty of Pharmaceutical Sciences, Dow University of Health Sciences, Karachi, Pakistan; ^6^Institute of Pharmaceutical Sciences, University of Veterinary and Animal Sciences, Lahore, Pakistan; ^7^School of Pharmaceutical Sciences, Universiti Sains Malaysia, Penang, Malaysia; ^8^Department of Pharmacy, King Abdullah Medical City, Ministry of Health, Makkah, Saudi Arabia; ^9^Pharmaceutical Care Department, King Abdulaziz Medical City, Ministry of National Guard - Health Affairs, Jeddah, Saudi Arabia; ^10^Internal Medicine Department, Alnoor Specialist Hospital, Makkah, Saudi Arabia; ^11^Department of Clinical Pharmacy, Faculty of Pharmacy, Al-Baha University, Al-Baha, Saudi Arabia; ^12^Department of Clinical Pharmacy, College of Pharmacy, Prince Sattam Bin Abdulaziz University, Al Kharj, Saudi Arabia; ^13^Department of Pharmacy Practice, College of Pharmacy, Umm Al-Qura University, Makkah, Saudi Arabia

**Keywords:** tuberculosis, diabetes mellitus, TB-DM comorbidity, multi-drug resistant TB, treatment failure, disease progression

## Abstract

**Background:**

The existence of Type 2 Diabetes Mellitus (DM) in tuberculosis (TB) patients is very dangerous for the health of patients. One of the major concerns is the emergence of MDR-TB in such patients. It is suspected that the development of MDR-TB further worsens the treatment outcomes of TB such as treatment failure and thus, causes disease progression.

**Aim:**

To investigate the impact of DM on the Emergence of MDR-TB and Treatment Failure in TB-DM comorbid patients.

**Methodology:**

The PubMed database was systematically searched until April 03, 2022 (date last searched). Thirty studies met the inclusion criteria and were included in this study after a proper selection process.

**Results:**

Tuberculosis-Diabetes Mellitus patients were at higher risk to develop MDR-TB as compared to TB-non-DM patients (HR 0.81, 95% CI: 0.60–0.96, *p* < 0.001). Heterogeneity observed among included studies was moderate (I^2^ = 38%). No significant change was observed in the results after sub-group analysis by study design (HR 0.81, 95% CI: 0.61–0.96, *p* < 0.000). In the case of treatment failure, TB-DM patients were at higher risk to experience treatment failure rates as compared to TB-non-DM patients (HR 0.46, 95% CI: 0.27–0.67, *p* < 0.001).

**Conclusion:**

The results showed that DM had a significant impact on the emergence of MDR-TB in TB-diabetes comorbid patients as compared to TB-non-DM patients. DM enhanced the risk of TB treatment failure rates in TB-diabetes patients as compared to TB-non-DM patients. Our study highlights the need for earlier screening of MDR-TB, thorough MDR-TB monitoring, and designing proper and effective treatment strategies to prevent disease progression.

## Introduction

The existence of Type 2 Diabetes Mellitus (DM) in tuberculosis (TB) patients causes serious public health concerns (1). People across different regions of the world are becoming affected by this comorbidity (2). The occurrence rate of DM in TB patients is rising globally and causes severe health problems in TB patients (3). DM affected more than 50% of TB patients across all regions of the world (4, 5). This co-morbid condition leads to many unfavorable TB treatment consequences such as treatment failure rates and increased drug resistance cases in TB patients (6, 7).

One of the challenges in controlling this TB-DM comorbid condition is the development of MDR-TB, in which resistance develops for both Isoniazid and Rifampicin (8). The presence of DM enhances the chances of developing MDR-TB and DM was recognized as a predictor for its emergence (9). The prevalence of MDR-TB ranged from 10 to 30% in diabetics (10, 11). The emergence of MDR-TB in TB-DM comorbid patients is very dangerous for the health of people (8). It can be the leading cause of death in people (8). The TB treatment cure rates have been reduced from 96 to 54% due to the presence of MDR in diabetic patients. The reason for reduced TB treatment cure rates and increased treatment failure rates may be due to complex treatment protocols and the adverse effects of alternative medications given to patients with MDR-TB (8). Moreover, it puts an increased financial burden on patients and becomes one of the reasons for disease progression (8, 12).

The “WHO Collaborative Framework for Care and Control of Tuberculosis and Diabetes” helps to guide healthcare professionals in the effective management of both diseases at administrative and clinical levels (13). The link between DM and MDR-TB is not well-studied in the literature. Only 13 studies were included in a previous systematic review and meta-analysis that reported the association between DM and MDR-TB and found that DM is a strong predictor to develop MDR-TB in TB patients and this TB-DM comorbid condition can potentially lead to high treatment failure rates (9). But to the best of the author’s knowledge, there is still a lack of studies on the association between DM and MDR-TB and TB treatment failure results. Moreover, there is also a need for adjusting potential covariates. A more comprehensive systematic assessment of the available literature is needed for further understanding of their association in TB-DM comorbid patients. Thus, this study’s goal was to investigate the impact of DM on the Emergence of MDR-TB and Treatment Failure rates in TB-DM comorbid patients versus TB-non-DM patients.

## Methodology

### Study design

The Preferred Reporting Items for Systematic Reviews and Meta-Analysis (PRISMA) guidelines were being followed by this study (14).

### Literature search

In this review, the PubMed database was systematically searched until April 15, 2023 (the last date of searching). For PubMed Search terms used were as follows: “Diabetes Mellitus” [Mesh]) OR “Diabetes Mellitus, Type 2” [Mesh]) AND “Tuberculosis” [Mesh]) OR “Tuberculosis, Pulmonary” [Mesh]) AND “Treatment Outcome” [Mesh]) AND “Drug resistance” [Mesh]) OR “Multidrug resistance” [Mesh]). The studies that were fulfilling the criteria were also searched from the references of the included studies. The review was only performed on English-language published papers.

### Inclusion and exclusion criteria

The inclusion criteria for this study are as follows: the research articles (case–control, cohort or cross-sectional) reporting the association of DM with both MDR-TB and treatment failure rates with adjusted covariates data, studies involving human subjects receiving anti-TB therapy, clinical outcome studies, studies investigating diabetes mellitus’s impact on tuberculosis treatment failure results in tuberculosis patients, and articles published in the English language. The study excluded non-research articles, non-human studies, reports, editorials, studies not analyzing the MDR-TB and treatment failure rates in patients, studies involving pre-DM and type-1 DM patients, and studies for which full text could not be retrieved. Three reviewers independently reviewed papers that fulfilled the selection process. If there were any conflicts between the reviewers, then those were clarified through discussion or the involvement of fourth reviewer.

### Study selection

The first step performed was to screen the studies by reading their title and abstracts. The entire content of the selected studies was further retrieved for additional review. For the evaluation of the quality of the individual research articles in the study and to assess the possible risk of bias among studies, the Newcastle-Ottawa Quality Assessment Scale (NOS) was used. The scale comprises of three parts: selection, comparability, and exposure (for cross-sectional or case–control studies) or measurement of outcome (for cohort studies). It is a nine-score quality assessment scale. A score of <3 means having poor quality and high risk of bias, ≥3 means having median quality and moderate risk of bias and a score of ≥7 means having high quality study and a low risk of bias (15).

### Data extraction

From the research articles included in this meta-analysis, the relevant information was collected using a data collection form. Author information, year, country of study, study design, participants, inclusion criteria, treatment regimen, covariates, TB treatment outcomes, and regression results with 95% CI and *p* values were the data of the included studies extracted. The relevant data were extracted independently by two reviewers. The data of included studies extracted were reviewed by a third reviewer. Any conflicts were sorted out through discussion until a final decision was taken.

### Statistical analysis

Heterogeneity among studies was assessed by using I^2^ test. The pooled HR (95% CI) were calculated using the fixed effects model. Subgroup analyses were performed by study design to assess the impact of study design on results. *p* < 0.01 was considered statistically significant. All statistical analyses were conducted by the meta program in STATA version 12.0 (Stata Corp, College Station, Texas).

## Results

### Search results

**Figure 1:**
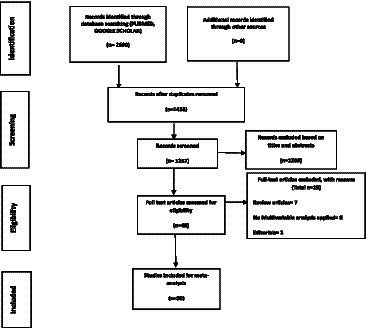
PRISMA flow diagram.

When searching the databases, 2,690 results were retrieved. A total of 1,257 articles qualified for the preliminary screening after 1,433 duplicates were removed. Based on their titles and abstracts, 1,208 studies were irrelevant to our study objective and were excluded. Out of 49 studies that were available for full-text review, 19 studies were excluded out of which seven studies were review articles, two studies were editorials, and eight studies did not use multivariable analysis for adjustment of covariates. Thirty studies were selected for inclusion in this meta-analysis after a complete article overview. Figure 1 depicts the selection process of studies included in the meta-analysis.

### Study characteristics

Among the included studies, 23 were cohort studies including 13 retrospective and 10 prospective and seven were case–control studies. There were six studies conducted in China (16–21), six in Mexico (22–27), three in South Korea (28–30), three in India (31–33) two in United States (25, 34), and one each in Armenia (35), Brazil (36), Spain (37), Indonesia (5), Bangladesh (38), Saudi Arabia (39), Ethiopia (40), Taiwan (41), Georgia (42), and Thailand (43). In the pool analysis, total number of included patients were 225,812, among which 180,619 were TB patients (ranged from 90 to 146,390), while 42,975 were TB-Diabetes comorbid patients (ranged from 24 to 34,988). Table 1 shows all the refined data of included studies.

**Table 1:** Summaries of the included studies.

Author	Year	Country	Research design	TB patients	Patients with DM comorbidity	Covariates	TB treatment outcomes	MDR in TB patients	MDR in TB-DM patients	*p* value
Sahakyan et al. (35)	2020	Armenia	Retrospective	585	36	Baseline weight, sputum smear status	Treatment failure, MDR-TB	90/585	5/36	0.809
Leung et al. (16)	2017	China	Prospective	18,083	3,331	Demographic factors, socioeconomic factors, medical factors, treatment-related factors, hepatitis, renal disease	MDR-TB			0.133
Lee et al. (30)	2017	South Korea	Retrospective	1,044	253	Gender, age, body mass index (BMI), cavitary disease, previous history of tuberculosis, and presence of diabetes mellitus	Treatment failure, MDR-TB	25/791	15/253	0.035
Mi et al. (17)	2014	China	Retrospective	434	187	Age, gender, occupation, resident area, and previous TB treatment	MDR-TB	-	10/26	<0.05
Siddiqui et al. (33)	2016	India	Prospective	266	37	Age, sex, BMI, TB history, risk factors, clinical presentation, treatment history, DM status, and adverse drug reaction	Treatment failure, MDR-TB	4/154	0	<0.05
Yoon et al. (29)	2017	South Korea	Prospective	157	108	Age, smoking status, uncontrolled diabetes, comorbidity, and positive sputum smear	Treatment failure, MDR-TB	18/401	6/94	0.466
Gomez-Gomez et al. (27)	2015	Mexico	Case–control	175	56	Age, gender, smoking, malnutrition, alcohol, and other underlying diseases	MDR-TB	39/139	17/36	<0.05
Gil-Santana et al. (36)	2016	Brazil	Retrospective	273	135	Age, gender	MDR-TB	3/244	12/128	0.002
Delgado-Sánchez et al. (26)	2015	Mexico	Retrospective	146,390	34,988	Gender, age, previous TB treatment, year of diagnosis, and malnutrition	Treatment failure, MDR-TB	630/1614	362/672	<0.001
Hongguang et al. (18)	2015	China	Prospective	944	182	Age, gender, and treatment categorization	Treatment failure, MDR-TB	4/944	2/182	0.238
Viswanathan et al. (32)	2014	India	Retrospective	149	96	Severity of Diabetes, glycemic control, and comorbidities	Treatment failure, MDR-TB	0	1/96	0.04
Nandhakumar et al. (31)	2013	India	Retrospective	2,449	667	Age, gender, type of TB, smear result, and HIV status	Treatment failure, MDR-TB	4/427	5/677	0.04
Fisher-Hoch et al. (25)	2008	USA	Retrospective	1,041	401	Age, gender, alcohol, drug abuse, HIV, and previous TB history	MDR-TB	31/1041	18/401	<0.05
Fisher-Hoch et al. (25)	2008	Mexico	Retrospective	1,149	287	Age, gender	MDR-TB	-	287/1436	<0.05
Suárez-García et al. (37)	2009	Spain	Case–control	655	41	Alcohol, age, and previous TB treatment	MDR-TB	27/30	3/30	<0.05
Jiménez-Corona et al. (24)	2013	Mexico	Prospective	888	374	Severity of disease, smear status	Treatment failure, MDR-TB	26/604	17/315	<0.001
Hsu et al. (19)	2013	China	Prospective	804	204	Age, gender	MDR-TB	24/665	5/204	0.926
Alisjahbana et al. (5)	2007	Indonesia	Prospective	540	94	Age, gender, BMI, and disease duration	MDR-TB	13/272	1/56	>0.05
Rifat et al. (38)	2014	Bangladesh	Case–control	917	83	Age group, education, occupation, and smoking	MDR-TB	-	-	0.001
Singla et al. (39)	2006	Saudi Arabia	Retrospective	505	187	Age, gender	MDR-TB	-	-	0.001
Bashar et al. (34)	2001	USA	Case–control	102	53	HIV, residency status	MDR-TB	-	-	<0.01
Adane et al. (40)	2023	Ethiopia	Prospective	243	24	Age, BMI, gender, smoking, and alcohol	Treatment failure, MDR-TB	1/132	3/60	0.056
Chiang et al. (41)	2015	Taiwan	Retrospective	768	705	Age, gender, sputum smear, drug resistance, and smoking	Treatment failure, MDR-TB	-	-	<0.01
Magee et al. (42)	2015	Georgia	Prospective	281	37	Age, gender, HIV infection, and smoking	Treatment failure, MDR-TB	32/229	11/37	<0.001
Munoz-Torrico et al. (23)	2017	Mexico	Retrospective	90	49	Age	Treatment failure, MDR-TB	34/41	39/49	0.7
Perez-Navarro et al. (22)	2017	Mexico	Prospective	324	183	Age, gender, overcrowding, and smoking	Treatment failure, MDR-TB	16/324	26/183	<0.001
Wu et al. (20)	2016	China	Retrospective	161	40	Age, gender, history of smoking, lung cavities, smear status, and TB treatment duration	MDR-TB	4/161	4/40	0.135
Min et al. (28)	2005	South Korea	Case–control	145	52	Age, smoking	MDR-TB	41/166	11/29	0.04
Song et al. (21)	2015	China	Case–control	900	54	Inadequate regimen, insufficient dose, and compliance	MDR-TB			<0.05
Jitmuang et al. (43)	2015	Thailand	Case–control	157	31	Age, gender, previous TB, HIV, alcohol, and smear status	MDR-TB	22/141	9/47	<0.001

DOTS, Directly observed treatment, short course strategy; NTP, National Tuberculosis Control Program; and WHO, World Health Organization.

### Statistical results

#### MDR-TB

**Figure 2:**
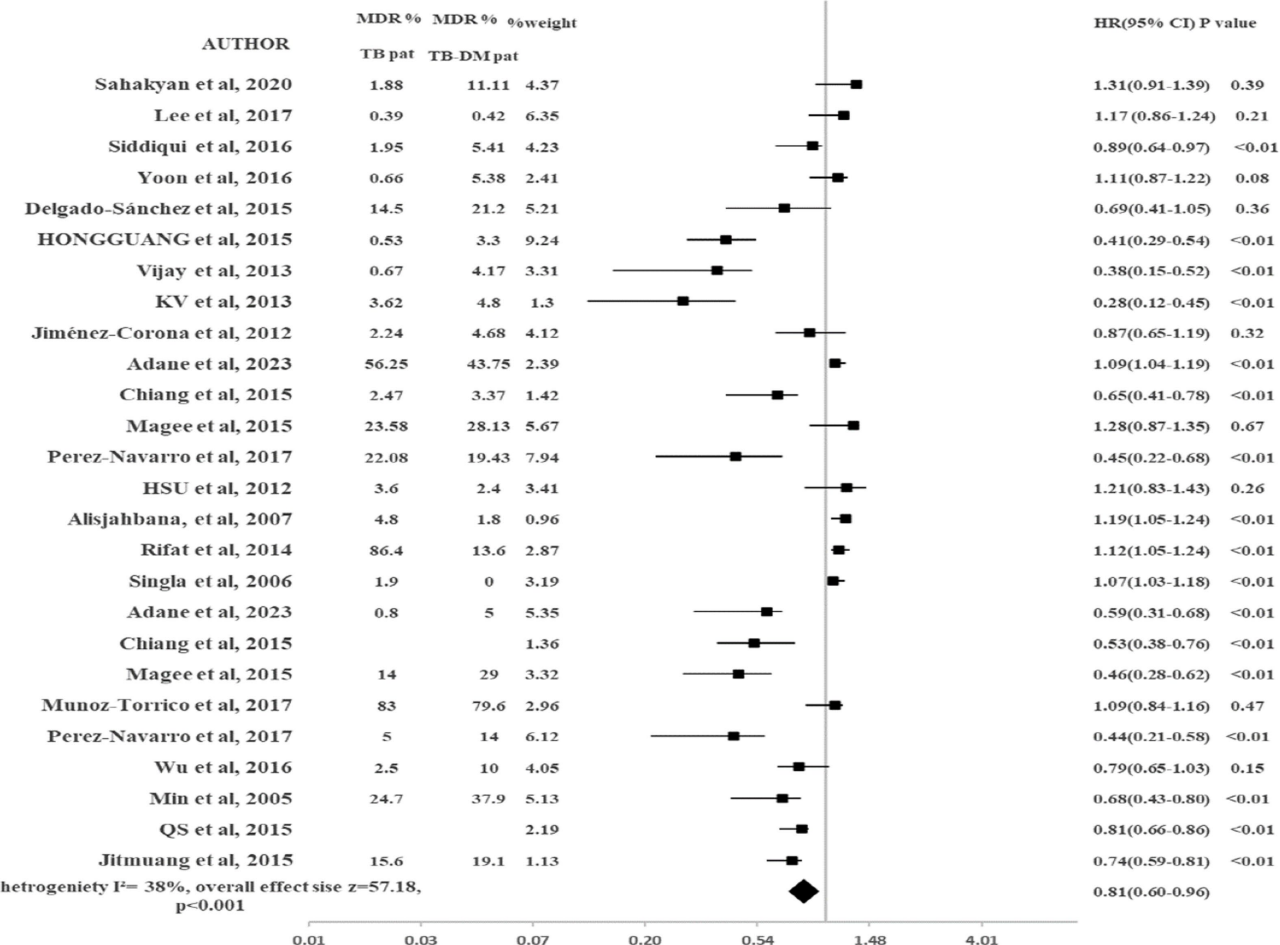
Impact of DM on development of MDR-TB.

**Figure 3:**
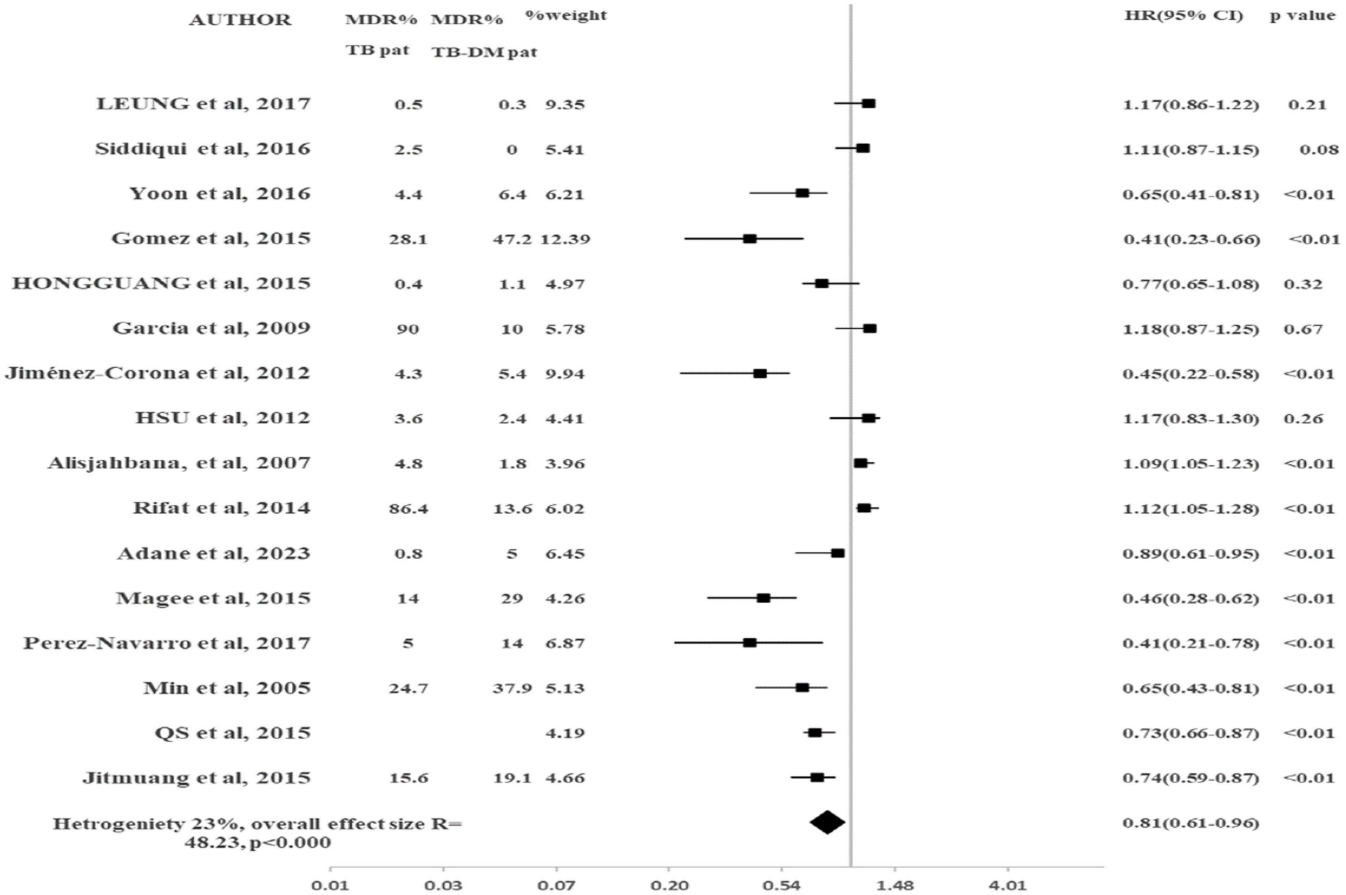
Sub-group analysis by study design.

The results of pooled data of MDR-TB for all included studies are shown in Figure 2. This study showed that TB-DM patients were at higher risk to develop MDR-TB as compared to TB-non-DM patients (HR 0.81, 95% CI: 0.60–0.96, *p* < 0.001). There was 19% reduced risk in TB-non-DM patients for MDR-TB emergence as compared to TB-DM patients. Heterogeneity observed among included studies was moderate (I^2^ = 38%). To check the impact of the study design on the results, the subgroup analysis was conducted. Only studies that were prospective were included in the sub-group analysis. No significant change was observed in the results (HR 0.81, 95% CI: 0.61–0.96, *p* < 0.000) as shown in Figure 2. The heterogeneity was reduced (I^2^ = 23%). The 95% CI was overlapping in both major analysis and subgroup analysis (Figure 3).

#### Treatment failure

**Figure 4:**
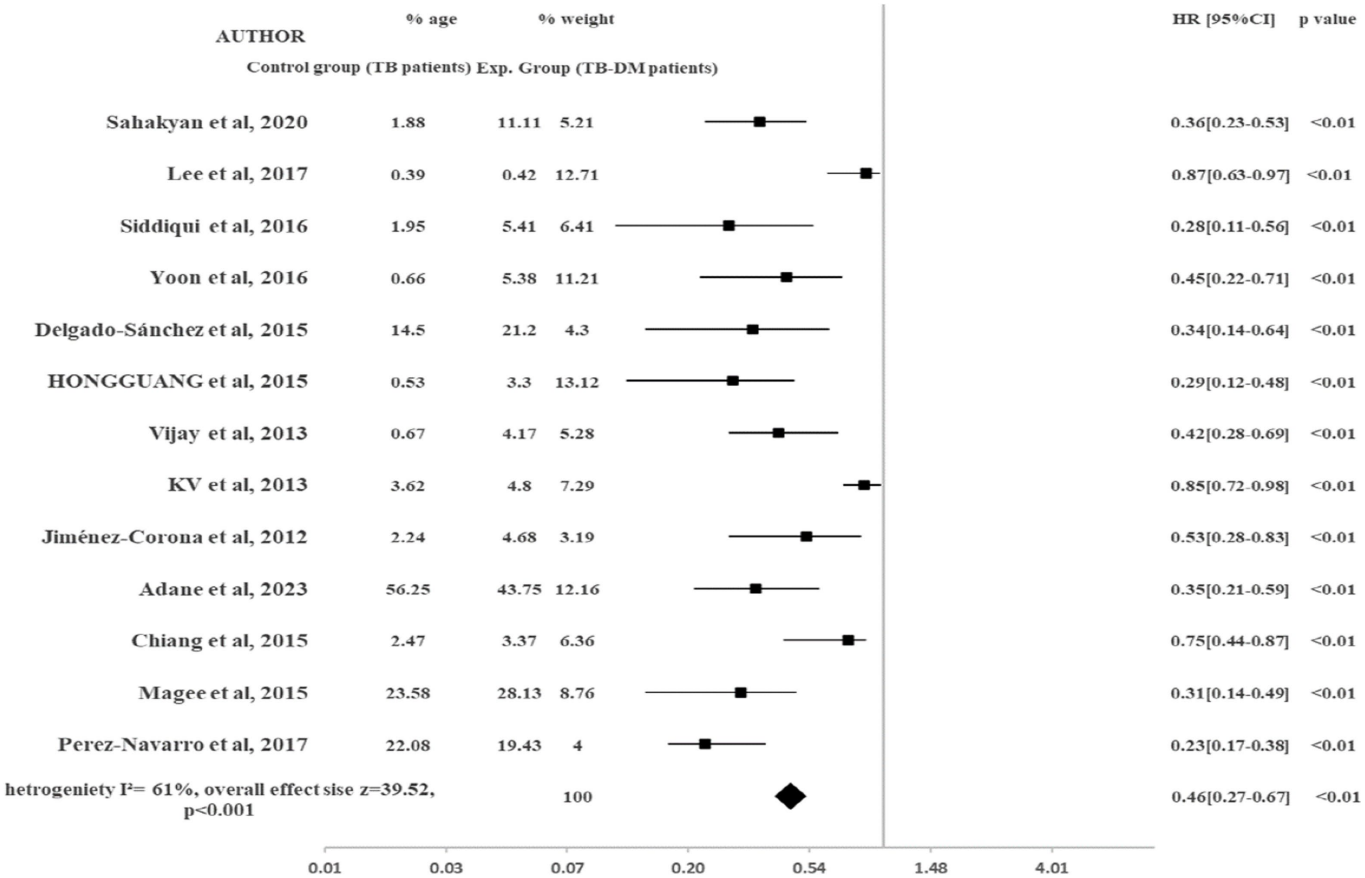
Impact of DM on TB treatment failure rates.

Figure 4 shows the impact of diabetes on treatment failure in TB-Diabetes comorbid patients. TB-DM patients were at higher risk to experience treatment failure rates as compared to TB-non-DM patients (HR 0.46, 95% CI: 0.27–0.67, *p* < 0.001). TB-non-DM patients were at 46% reduced risk for experiencing treatment failure rates as compared to TB-DM patients. The heterogeneity observed among included studies was (I^2^ = 61%).

### Risk of bias assessment

The included studies showed NOS score range of 6–9. Out of 30 studies, the quality of 21 studies was high. The criteria for selection domain were fully met by all studies except five studies. The age factor was not adjusted in multivariable analysis in five studies in section of confounding bias. For bias in exposure measurement, the non-response rate was not reported in seven studies by the cases as well as controls. The overall NOS mean score of included studies was seven, indicating low biasness risk and high quality of the included studies. Table 2 provides the risk of bias among included studies.

**Table 2:** Risk of bias among included studies.

Author, Year	Research design	Selection	Comparability	Outcome/Exposure	NOS score	Risk of Bias
Sahakyan et al. (35)	Retrospective	****	*	*	6	Moderate
Leung et al. (16)	Prospective	****	**	***	9	Low
Lee et al. (30)	Retrospective	****	*	*	6	Moderate
Mi et al. (17)	Retrospective	****	**	***	9	Low
Siddiqui et al. (33)	Prospective	***	**	*	6	Moderate
Yoon et al. (29)	Prospective	****	**	*	7	Low
Gomez-Gomez et al. (27)	Case–control	****	**	**	8	Low
Gil-Santana et al. (36)	Retrospective	****	**	*	7	Low
Delgado-Sánchez et al. (26)	Retrospective	****	**	*	7	Low
Hongguang et al. (18)	Prospective	****	*	*	6	Moderate
Viswanathan et al. (32)	Retrospective	***	*	**	6	Moderate
Nandhakumar et al. (31)	Retrospective	****	**	***	9	Low
Fisher Hoch et al. (25)	Retrospective	****	**	**	8	Low
Fisher Hoch et al. (25)	Retrospective	****	**	**	8	Low
Suarez Garcia et al. (37)	Case–control	****	**	**	8	Low
Jiménez-Corona et al. (24)	Prospective	****	*	**	7	Low
Hsu et al. (19)	Prospective	****	**	***	9	Low
Alisjahbana, et al. (5)	Prospective	***	*	**	6	Moderate
Rifat et al. (38)	Case–control	****	**	**	8	Low
Singla et al. (39)	Retrospective	****	**	***	9	Low
Bashar et al. (34)	Case–control	****	*	*	6	Moderate
Adane et al. (40)	Prospective	***	**	***	8	Low
Chiang et al. (41)	Retrospective	****	**	***	9	Low
Magee et al. (42)	Prospective	****	**	***	9	Low
Munoz-Torrico et al. (23)	Retrospective	****	*	**	7	Low
Perez-Navarro et al. (22)	Prospective	***	**	*	6	Moderate
Wu et al. (20)	Retrospective	****	**	*	7	Low
Min et al. (28)	Case–control	****	**	*	7	Low
Song et al. (21)	Case–control	****	*	**	6	Moderate
Jitmuang et al. (43)	Case–control	****	**	*	7	Low

## Discussion

The TB-DM comorbidity has become more prevalent worldwide and it has become a major challenge for healthcare organizations to control this comorbid condition (44). As it is understood that diabetes in TB patients influences negative TB treatment outcomes including sputum positivity, high risk of relapse, and mortality (17, 39). It is a matter of concern that the development of MDR-TB in TB-DM comorbid patients may further worsen the TB treatment outcomes. It puts an additional financial burden on patients that cause disease progression and ultimately leads to the extensively drug-resistant TB (XDR-TB) (45). This study showed a significant association between DM and MDR-TB.

In this study, there was a lower risk of MDR-TB emergence for TB-non-DM patients when compared with TB-DM comorbid patients (HR 0.81, 95% CI: 0.60–0.96, *p* < 0.001). Our results were found comparable to the previous studies and meta-analyses that found a significant association between DM and MDR-TB (9). The following factors can be the reason for the emergence of MDR-TB: (1) Due to DM, impaired immune functioning, altered microbial genomics, and uncontrolled glucose levels can enhance the susceptibility to develop MDR-TB in TB patients. (2) DM may also enhance the risk of MDR-TB emergence in TB patients due to DM include phagocytotic activity, chemotactic response, generation of oxidative species, microbe proliferation, altered drug disposition, and non-adherence to treatment (46–50). While results were inconsistent with few previous studies (5, 27, 30–32, 51). The limited number of the patients, geographical variations, and unadjusted covariates may be the reason for these discrepancies in results and should be further addressed.

Our study showed a lower risk of experiencing treatment failure rates in TB-non-DM patients as compared to TB-DM comorbid patients (HR 0.46, 95% CI: 0.27–0.67, *p* < 0.001). Diabetics are more prone to TB treatment failure rates. The TB treatment failure results in our study were found similar with previous studies (51–53) but discrepancies in results were found in the previous three studies (30, 39, 51). The small sample size and study design used in studies may be the factors causing discrepancies in results. However, most of the studies in our study assessed by the NOS scale were of high quality and there was a lack of lab data for acquiring information on risk factors.

Most of the information was based only on self-reporting or health records of patients, which can lead to assessment error. There should be complete reporting of non-response rates among cases as well as controls to assess reporting-based bias properly. There was moderate heterogeneity across all studies. We performed subgroup analysis by study design, the heterogeneity was reduced. With sub-group analysis by study design for MDR-TB, no change in results was noted when we included prospective studies (HR 0.81, 95% CI: 0.61–0.96, *p* < 0.000).

There are some limitations present in our study. The studies with different study designs were included that may lead to heterogeneity in results. The heterogeneity in control groups can affect the association to be determined. We assessed overall MDR-TB patients not by primary and secondary subtypes, which may cause differences in risk factors and outcomes. Moreover, there may be the possibility of publication bias, even though the study included a comprehensive set of studies. The statistical techniques used to address the variability in the study design may introduce some uncertainty in the findings. Despite few limitations, the study has several strengths. The main findings of this study given us a valuable understanding of how DM affects emergence of MDR-TB in TB-Diabetes comorbid patients. The strengths of our results lie in comprehensive analysis of a significant number of studies, focusing on a specific patient population. We focused on specific subtypes of DM and TB, providing a more refined understanding of their interaction. The screening for MDR-TB should be prioritized at an earlier stage in TB-DM comorbid patients, which could reduce the economic burden on these patients. There should also be thorough MDR-TB monitoring and necessary steps should be taken to design a proper treatment strategy to prevent the disease progression in these patients. For future concern, this study highlights the importance of conducting more thorough research with large groups of people, using consistent methods, and considering variables that might affect the results. This would help gain a better understanding of how Type 2 DM affects TB-DM comorbid patients.

### Conclusion

The results showed that DM had significant impact on emergence of MDR-TB in TB-Diabetes comorbid patients as compared to TB-non-DM patients. DM enhanced the risk of experiencing TB treatment failure rates in TB-Diabetes patients as compared to TB-non-DM patients. Our study highlights the need for earlier screening of MDR-TB, thorough MDR-TB monitoring and designing proper and effective treatment strategies to prevent disease progression.

## Author contributions

MK, AR, UM, ML, IA, MR, SS, KH, RH, GA, AA, MA, SAlg, MI, SAlm, SK, and AH made substantial contributions to the conception and design of the study, and analysis and interpretation of the data, and drafted the work or revised it critically for important intellectual content. MK and AR made substantial contributions to the analysis and interpretation of the data. All authors contributed to the article and approved the submitted version.
